# Relationship between bases of power and job stresses: role of mentoring

**DOI:** 10.1186/2193-1801-3-432

**Published:** 2014-08-13

**Authors:** May-Chiun Lo, Ramayah Thurasamy, Wei Tak Liew

**Affiliations:** Faculty of Economics and Business, Universiti Malaysia Sarawak, 94300 Kota Samarahan, Sarawak, Malaysia; School of Management, Universiti Sains Malaysia, 11800 Georgetown Pulau, Pinang, Malaysia

**Keywords:** Power bases, Mentoring, Job stresses, Malaysia

## Abstract

Building upon the social exchange theory, this paper hypothesized the direct effect of bases of power on job stress with mentoring as moderator. Power bases and job stresses were conceptualized as 7- and 3- dimensional constructs, respectively. One hundred and ninety-five Malaysian managers and executives working in large-scale multinational companies participated in this study. The results have indicated that bases of power as possessed by supervisors have strong effect on employees’ job stress and mentoring was found to have moderated the relationship between power bases and job stress. Implications of the findings, potential limitations of the study, and directions for future research were discussed further.

## Introduction

Leadership is a power relationship that exists between leaders or followers (Northouse 2001) and a process which involves utilizing power to influence the behaviours of others to meet the organizational goals (Flynn et al. 2012; Patrick 2012). Leadership cannot take place without the participation of the subordinates and power is the essence of leaders’ behavior. Kanungo (1998) regarded leadership as exercising influence over others by utilizing various bases of social power in order to achieve organizational objectives. Recent research studies have elucidated that power can be transformed into various forms and the important of its existence in the present organizations (Griffin and Van Fleet 2014).

Researchers in the past (e.g., Cooper and Dewe 2004; Makhbul and Khairuddin 2013) have been debating on the usage of the term “stress”. The earlier social psychological stressors to be studied and dominated the early history of work stress are known as role ambiguity and role conflict. They have been viewed as critical elements for two decades and are probably still the most frequently measured causes of work stress (Cooper and Dewe 2004). Sources of job stress comprise the organizational climate caused by the leadership style of supervisors. Some researchers have suggested that the research of work-related stress should comprise variables that show the organizational norms and expectations operating within the workforce, like leadership style and the lacking of ability of employees to apprehend the demands of his or her job (e.g. Ryska 2002; Stoppler 2011).

As indicated by past researchers, Malaysians suffering from job stress is not a new issue (Aniza et al. 2010; Myrtle et al. 2010). There are many factors contributed to job stress. Job stress emerges when people face circumstances that they appraise as taxing or surpassing their resources and endangering their well-being (Lazarus and Folkman 1984; Rani et al. 2013; Ramezani et al. 2013). A politically-charged workplace is one of such circumstances. Employees cannot be certain that their efforts will be rewarded or have confidence that they will not be put at risk by the actions of others when a workplace is politically charged. This unpredictable, risky, and threatening workplace context increases the level of job stress experienced (Cropanzano et al. 1997) for those who are not able to evade such contexts as well as those who have decided to join in the politicking. Stress can also exist if the employees feel “under loaded” through lack of stimulus or social contact. Other job stress contributors comprise role ambiguity, conflicting performance expectation, and poor relationships with other co-workers; social economics, and family matters (Manshor et al. 2003; Dar et al. 2011).

Besides, job stress will also appear because of the relationship of individuals with their mentors. Erkutlu and Chafra (2006) hypothesized that perceived supervisory position powers, which are legitimate, reward, and coercive power would be positively related to subordinate stress because they are likely to evoke a lack of personal control at work. Not solely is the subordinate highly dependent on the supervisor (Emerson 1962), the administration of the reward or punishment by the supervisor also lies beyond the subordinate’s direct control. Subordinate stress is likely to be provoked by the perceived lack of control and the anxiety associated with the need to satisfy the supervisor (Elangovan and Xie 2000). For that reason, perceived supervisor reward and coercive power will be positively connected to subordinate stress. Similarly, because the subordinate is reminded of responsibilities to be fulfilled and realizes that his or her performance will be monitored and evaluated, perceived legitimate power of the supervisor would be positively related to stress. Subordinate stress will likely be increased by the constant focus on duties and evaluation.

In response to these potential problems, many forward-thinking organizations are striving to create a positive organizational climate in order to keep those good employees through various human resource management initiatives (Chew and Chan 2008). While a great deal of past researches was done to investigate the link between power bases and job stress, relatively few researches have been conducted to examine these two components with the presence of mentoring effect. It is important for the company to know what aspects play important roles or have big effect in reducing the job stress of the employees. Moreover, there is a noticeable lack of empirical examination of large Malaysian organizations with regards to the leaders’ power bases on job stress. Hence the purpose of this study is two folds. First, to investigate the direct relationship between power bases of supervisors on employees’ job stress, and secondly to examine if mentoring will moderate the relationship between power bases and job stress.

## Literature review

Extensive research is available in the organizational behavior literature investigating the process of job stress and social power. But the two constructs—job stress and power bases seem to have been examined almost independently. That is, little research has been done to examine the relationship between bases of power and employees’ job stress in organizations. This is particularly true in the Malaysian context. This section is further divided into various sections in order to sequentially discuss the vital literature for each component that creates the foundation of this research.

### Power bases

In broadest terms, power has to do with getting things done, or getting others to do them. Social power exists when people with differing levels of potential power interact to accomplish the goals of the organization. Several experts (Emerson 1962; Fiske 1993; Thibaut and Kelley 1959) stated that power is the ability to provide, withhold resources or administer punishment, and with reference to a particular relationship or group. This is mainly because less powerful individuals feel that power holders will be able to help them to achieve their objectives. Tjosvold et al. (2003) noted that the tendency of leaders to consider power as limited is related to the traditional definitions of power where theorists have defined power in terms of getting others to do what they want them to do despite their resistance (Kipnis 1976; Weber 1947). Anderson and Berdahl (2002) in analyzing past psychological literatures on power found power to be organized around three main issues: the motive to attain power, the bases of power, and the consequences of having power.

Past literatures have revealed that bases of power are often interdependent, used in combinations or overlapped with one another. As Rodrigues (1995) argued, some of the power bases have similar characteristics especially when it is from an attribution point of view, an example would be personal or positional power. This is further supported by Lawrence et al. (2005) that there should be a balance among the various types of power in an organization in order to manage exploitation or exploration of tension efficiently and further concurred that the target of influence and the power relationship between the individuals would affect the job stress level of the employees. (Munir et al. 2012). Power in organizations exists together as a result of an individual’s position in a time and place and also due to his or her personal qualities (Hollander and Offermann 1990). Although some researchers have categorized power into two dimensions according to their own definition, it is still generally grouped under the umbrella of position and personal power. Position power is defined as having a certain degree of power inherent in its position in the organization, such as legitimate, reward, and coercive power (Bass 1960; Ragins and Sundstrom 1989; Rodrigues 1995; Rodrigues and Lloyd 1998; Gibson et al. 2012; Meng et al. 2014); whereas, personal power refers to the potential influence based on one’s expertise, charisma, and approachability such as expert, referent, connection, and information power (Rodrigues and Lloyd 1998; Lunenburg 2012).

### Job stress

Stress had been the object of examination in medicine, organizational psychology, engineering, organizational behaviour and many other discipline. The multidisciplinary nature of stress study has led to many meanings. In spite of that, agreement can be discovered in the definition of stressors and strains. Job stressors indicate that work circumstances or features are the cause to a person’s stress. Strains usually refer to physiological, behavioural, and psychological responses to stressors (Beehr 1984). Researchers in the past have highlighted that work characteristics or stressors (e.g. work overload, skill underutilization) can lead to individual perceptions of stress according to the general work-stress health model (House 1981; Katz and Kahn 1978; Shukla and Garg 2013). Individual factors such as occupation, education, sex, and experience that may act as potential moderators of stress, influence these perceptions (Ivancevich and Matteson 1980). Job-related strains (e.g. higher blood pressure, anxiety, and intent to quit) trigger individual perceptions of stress. The more or less lengthy feeling of strains may affect the person’s health (e.g. sleep loss, coronary heart disease, and alcohol abuse) and may also have indirect effects on the person’s on the job performance. Strains can also be led by lack of control (Thompson 1981). The following sections are some components of job stress.

### Role conflict

Role conflict can be resulted by trying to meet the demands of two or more groups (i.e. customers and managers) at the same time (Rizzo *et al.*^1970^). Effect on job performance is an important consequence of role conflict. Flaherty et al. (1999) found that role conflict is negatively related to customer-oriented selling, a trait associated with increased job performance in a study of salespeople representing various industries. Nevertheless, psychological withdrawal from the job leading to reduced job performance may be experienced by employees encountering role conflict (Bettencourt and Brown 2003).

However, results of studies investigating the effects of role conflict on job performance have been inconsistent. For example, some researchers (Bhuian et al. 2005; Lusch and Jaworski 1991; Singh 1998; Ahmad 2008) discovered that role conflict had a negative consequence on job performance, and others (Babin and Boles 1996; Dubinsky et al. 1992) In addition to that, role conflict happens when the behaviours expected of an individual are inconsistent according to role theory. If the expectations are not achieved, this person will suffer stress, become dissatisfied, and perform less effectively. Role conflict exists from two very unlike policies or insistent requests and produces individual dissatisfaction and decreased organizational performance Rum et al. 2013; Vanishree 2014). As evidenced in the past, most researchers consent that utmost role conflict will gradually destroy job performance gradually (Singh et al. 1994; Smith 2011; Karimi et al. 2014).

### Role ambiguity

Role ambiguity refers to the degree to which clarity is lacking in the anticipations connected with a role, in the ways for carrying out known role anticipations or in the results of role performance (Kahn *et al.*^1964^). It can also be described as a deficiency of information required to perform the role (Cooper et al. 2001). Other researchers defined role ambiguity as the absence of clarity and predictability in the job (Menon and Akhilesh 1994). Role ambiguity will result in coping behaviour by the uncomfortable employees in organizations who may attempt to solve the problems for avoiding stress, or use defence mechanisms for changing the real situation according to role theory. For that reason, ambiguity will permit an employee to be dissatisfied with his role in the organization, alter reality, and reduce his performance.

Similarly, role ambiguity or a lack of role clarity (Shepherd and Fine 1994) means a lack of understanding about job responsibilities and knowing what is expected in terms of one’s job performance. Employees who are experiencing role ambiguity tend to have lower performance (Bhuian *et al.*^2005^) than employees who have a complete understanding of job instructions and what are expected of them (Babin and Boles 1998; Karimi et al. 2014). Customer-oriented behaviour and, ultimately, profitability can be constrained if experiencing role ambiguity (Flaherty *et al.*^1999^). Wetzels et al. (2000) found that role ambiguity and a commitment to delivering service quality are negatively related in a study of retail salespeople. Ambiguity is especially serious in those functional areas where managerial positions inclined to be less concrete in nature and need more abstract thinking and decision making. Such uncertainties may emerge because the anticipations describing the role are themselves ill-defined and not consistent.

### Role overload

Role overload can be described as an individual lacks of resources to satisfy distinct roles, where there is a need to apply commitments, obligations, or requirements (Peterson *et al.*^1995^). Being the most frequently mentioned stress within the three sources, role overload can be viewed as the amount of work to be done within a given period of time and lead to over demands of working time and create uncertainty of performance (Cooper *et al.*^2001^; Cooper and Dewe 2004).

When the work requires skills, abilities, and knowledge beyond what the person has, qualitative overload occurs. In addition, qualitative overload occurs when employees feel that they are lacking of the ability to do the job regardless of the amount of time available to them to complete the job. It may also arise when performance standards are fixed so high as to appear not attainable (Larson 2004).

### Psychological discord

In a survey of job stress and its effect at an university, Dua (1994) found that psychological distress is among the factors that contribute to higher levels of job stress. Psychological stress is likely to be expressed as psychological symptoms including sleep disturbances, anxiety, panic attacks, dysphoria, and restlessness (Edwards et al. 1998). Behavioural changes of the kind frequently monitored in stress management interventions such as increased absenteeism (Murphy and Sorenson 1988), insurance claims, and use of health care services may be resulted by this stress.

### Lack of control

Lack of control also constitutes a source of stress (Karuppan 1995). Perceived lack of control (Perrewe and Ganster 1989) has been recognized as work-related stressors. More exactly, this line of study shows that stress arises when employees perceive a lack of sufficient control over deciding how to perform their task. Kaldenberg and Becker (1992) tested the relationship between the amount of workload preferred and the real amount undergone. They concluded that workers with greater control and autonomy undergo less strain. Hence, lack of control can be viewed as organizationally-induced stressors.

### Relaxation

Relaxation is a kind of meditation, a state of concentration. One cancels out all distraction associated with everyday life by using the mind to focus upon an object, image, or thought. The “relaxation response” is induced to counter balance the stress response. The “relaxation response” has four basic elements (Ross and Altmaier 2000), which are a quiet environment, a comfortable position, an object, thought, or image to dwell upon, and a passive attitude. The greater probability of managerial stress and greater consciousness of the issue of legal liability has, understandably, cause problem to increasing consideration. As a new breed of stress consultants become recognized, the means of measuring stress in organizations are developed. Although those strategies concentrate on symptoms and by creating the illusion that something positive are being done, they actually inhibit the identification and tackling of the basic causes of stress (HSE 1993, 1995).

### Mentoring

Most research describes only two or three functions of mentors although Burke’s (1984) research demonstrated almost 15 different functions. As stated by Kram (1985), a mentor provides support, direction, counselling, friendship, advice, increased employee exposure and visibility towards career development. These functions can be simplified to two roles, which are career and psychosocial support. Three different functions distinguished by Gregory et al. (2006), which are career development, psychosocial support (Allen and Eby 2004), and role-modelling (Burke 1984; Scandura 1992; Scandura and Viator 1994; Wallace 2001). Psychosocial support and role modelling are frequently joined together in literature (Gregory et al. 2006). As stated by Allen and Eby (2004) and Elliott et al. (2007), female mentors offer more psychosocial mentoring than do male mentors, who offer more career mentoring supports.

Mentorship contributes to improved employee motivation, performance, commitment, and retention. Successful mentorship assists the progress of leadership development and can be an effective means of recognizing talent. As mentors relay norms and values (Wilson and Elman 1996), organizational culture and philosophy can be promoted. Mentoring also develops human resources by operating as a kind of on-the-job training, which results in building a competent workforce (Allen *et al.*^1997^). The following sections are dimensions of mentoring which are adopted in this study.

### Career support

Career support involves coaching, sponsorship and protection, and is associated with increased exposure and visibility, facilitating advancement career in the organization and satisfaction (O’Neill 2002). Mentors offer young adults with career-enhancing functions, like sponsorship, coaching, acilitating exposure and visibility, and offering challenging work or protection, all of which help the younger individual to set up a function in the organization, learns the ropes, and prepare for advancement (Kram and Isabella 1985). The “career-enhancing functions” that exist in a mentoring relationship are concentrated on the organizational context; that is, they intensify a person’s capacity to secure jobs, to cause improvement in the organization, and to develop expertise that are essential to satisfactory workplace performance and promotion (Ritchie and Genoni 2002).

Jennings (1971) found that most corporate presidents had mentors and that the mentoring process was vital to their achievements, in regards to the career-development function. Roche’s research (1979) states that among the important business executives in major U.S. corporations tested, those who had mentors usually accepted higher salaries, bonuses and total compensation than their counterparts who did not have mentors.

### Psychosocial support

Psychosocial support is another dimension of mentoring where mentor offers role modelling, counselling, confirmation, and friendship in the psychosocial sphere, which help the young adult to develop a sense of professional identity and competence (Kram and Isabella 1985). The “psychosocial functions” are those which support a person’s sense of self-esteem and belief in their capacity to work effectively in their chosen profession (Ritchie and Genoni 2002). Levinson *et al.*’s (1978) study of male professionals showed that mentoring is the most important element of their psychosocial development, while Henning and Jardim (1977) reached the same conclusion about the importance of mentoring for women, in regards to the psychosocial benefits.

### Hypotheses

Erkutlu and Chafra (2006) hypothesized that perceived supervisory legitimate, reward, and coercive power would be positively related to subordinate stress with regard to position powers because they are likely to evoke a sense of lack of personal control at work (Costa and Martins 2011; Lees 2014). The administration of the reward or punishment by the supervisor also lies beyond the subordinate’s direct control, not only is the subordinate highly dependent on the supervisor (Emerson 1962; Erkutlu et al. 2011; Munir et al. 2012). Subordinate stress is likely to be provoked by the perceived lack of control and the anxiety associated with the need to satisfy the supervisor (Ganster and Schaubroeck 1991; Kahn and Byosiere 1992; Elangovan and Xie 2000). For that reason, perceived supervisor reward and coercive power will be positively connected to subordinate stress. Similarly, because the subordinate is reminded of responsibilities to be fulfilled and realizes that his or her performance will be monitored and evaluated, perceived legitimate power of the supervisor would be positively related to stress. Subordinate stress will likely be increased by the constant focus on duties and evaluation. Hence the following hypothesis is developed.

#### H1: Positional power bases of the mentor is positively related to subordinate stress

On the other hand, Erkutlu and Chafra (2006) hypothesized that expert and referent power of the supervisor has a negative relationship with subordinate stress (Munir et al. 2012). Perceiving one’s supervisor to be high on expert and referent power can be seen as similar to having a powerful social support system at work where the subordinate would consider the supervisor’s expertise to be source of work support whereas the supervisor’s personal appeal and likeability would produce a sense of interpersonal support. The significant benefits of having strong social support in dealing with stress had been noted by several researchers (e.g. Cohen and Wills 1985; Kahn and Byosiere 1992). Previous study has demonstrated that expert power and referent power are positively correlated with subordinate affect (Podsakoff and Schriesheim 1985), and expert power is negatively connected with subordinate job tension (Sheridan and Vredenburgh 1978). Thus, hypothesis 2 was established as follows.

#### H2: Personal power bases of the mentor is negatively related to subordinate stress

Kram (1985) identified four phases in the mentor-protégé relationship. They are initiation, cultivation, which is the heart of the mentoring relationship; separation, redefinition, at the end of the process. Purcell (2004) suggested that “even mentors need mentors at times” or at least someone with whom they can talk about mentoring issues. Past studies have posited that protégés who received greater psychosocial and career support showed greater stress reduction (Fullick et al. 2012). In addition to that, mentors hold the leadership power while protégés facing job stress by their mentors. Therefore, the researcher believed that mentoring will moderates the relationship of power and job stress where mentoring acts as a moderator to both mentors and protégés and as stated in hypothesis 3.

#### H3: Mentoring will moderate the relationship of power and job stress

**Figure 1:**
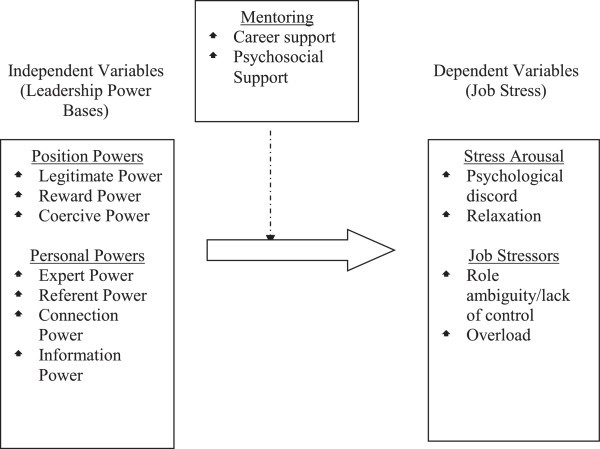
**Theoretical framework.** *Note:* Block arrow indicates direct effect; broken line indicates moderator.

The framework as shown in Figure 1 consists of two main variables, which are the independent variable and the dependent variable. The independent variables consist of seven bases of power, which are legitimate power, reward power, coercive power, expert power, referent power, connection power, and information power. The dependent variables – the four job stresses are also divided into stress arousal and job stressors. The moderator of mentoring, which consists of career support and psychosocial support have been included to boost up the framework.

## Methodology

### Research design, sample, and procedure

This study focuses on bank employees in Malaysia as a population of interest. Currently, the banking sector is considered as one of the cornerstone of Malaysia’s economic diversification strategy. The researcher has selected the large scale banks in Kuching using non-random sampling. A total of 250 questionnaires were distributed personally to the human resource managers in 10 banks. The large number of questionnaires given out was to ensure a sufficient number of returned questionnaires. Out of the 250 questionnaires, only 195 questionnaires were used for analysis. Initially, the researcher visited the selected banks to get the approval from the banks to conduct the survey. The questionnaires, together with the cover letters seeking their cooperation and explaining the purpose of the study as well as self-addressed envelopes for the completed questionnaires were all personally handed to the staffs after a brief personal communication concerning the topic and the goals of the study.

The measuring instrument for data collection from the supervisors was in the form of questionnaires and was divided into three sections. Section 1 required the respondents to rate a total of 35 items on their supervisors’ bases of power. The five bases of variables, namely legitimate, reward, coercive, expert, and referent power proposed by French and Raven were measured by using Hinkin and Schriesheim’s (1989) method. In addition, the items for another two power bases, namely information and connection, were taken from the work by Ansari (1990). Section 2 required respondents to assess their job related stress was assessed via the Job-Related Tension Index (JTI), a 10-item, 7-option scale developed by Kahn *et al.* (1964), which has been utilized extensively to assess organizational stress (Burke 1976). Section 3 was used to measure the two supports employed by mentors in their attempts to support their mentees. Those items used as supports were not collected from a single source, but were gathered from different studies (Kram 1985; Russell and Adams 1997). Finally, Section 4 was used to collect the personal profile and demographic data of respondents.

## Data analysis

### Profile of the respondents

Table 1 shows the demographic profile of the respondents. Based on Table 1, male respondents were more than female respondents with 106 male respondents (54.4%) and 89 female respondents (45.6%). Of the 195 respondents, majority were Chinese (83 or 42.6%), followed by others (63 or 32.3%), Malay (28 or 14.4%), and Indian (21 or 10.8%). The majority of the respondents were classified within 31 – 40 years old (71 or 36.4%), followed by 21 – 30 years old (54 or 27.7%), 41 – 50 years old (42 or 21.5%), below 21 years old (16 or 8.2%), and 51 years old and above (12 or 6.2%). The biographical details were as shown in Table 1.

**Table 1:** **Demographic profile of respondents**

		Respondents (***N*** = 195)	
Demographic variable	Category	Frequency	Percentage
Gender	Male	106	54.4
	Female	89	45.6
Race	Malay	28	14.4
	Chinese	83	42.6
	Indian	21	10.8
	Others	63	32.3
Age	Below 21 years old	16	8.2
	21 – 30 years old	54	27.7
	31 – 40 years old	71	36.4
	41 – 50 years old	42	21.5
	51 years old and above	12	6.2
Educational level	SPM	53	27.2
	STPM	45	23.1
	Diploma	58	29.7
	Degree	39	20.0
Position	Clerical staff	133	68.2
	Head	27	13.8
	Manager	35	17.9
Income per month	< RM 1500	22	11.3
	RM 1501 – RM 3000	83	42.6
	RM 3001 – RM 4500	55	28.2
	RM 4501 – RM 6000	23	11.8
	> RM 6001	12	6.2

### Goodness of measures

To verify the psychometric attributes of the instruments used in this research, different examinations were first conducted, followed by factor analysis.

The 18 items of mentoring components and 35 items of power bases were subject to a varimax rotated principal component analysis and were subsequently resulted in two and five interpretable factors, respectively with eigenvalues greater than 1. In total, the two factors of mentoring and five factors of power bases explained a total of 77 percent and 80 percent of the variance as shown in Tables 2 and 3, respectively. The 2 extracted factors of mentoring with factor loadings that ranged from .636 to .889 were subsequently renamed. Factor I which included 11 psychosocial support items was named as “psycho”. Factor II which included 6 career support items was named as “career”.

**Table 2:** **Mentoring relationship: rotated factors, item loadings, and reliabilities**

Items	Factors	
	I	II
**Factor I: Psychosocial support**		
Encouraged you to talk openly about anxiety and fears that detract from your work	**.889**	
Shared personal experiences as an alternative perspective to your problems	**.847**	
Conveyed empathy for the concerns and feelings you have discussed with him/her	**.843**	
Encouraged you to try new ways of behaving on the job	**.832**	
Conveyed feelings of respect for you as an individual	**.827**	
Encouraged you to prepare for advancement	**.793**	
Displayed attitudes and values similar to your own	**.791**	
Discussed your questions or concerns regarding feelings of competence, commitment to advancement, relationships with peers and supervisors or work/family conflicts	**.783**	
Given or recommended you for assignments that helped you meet new colleagues	**.727**	.500
Shared history of his/her career with you	**.705**	.561
Kept you informed about what is going on at higher levels in the company or how external conditions are influencing the company	**.699**	
Protected you from working with other managers before you knew about their likes/dislikes, opinions or controversial topics and the nature of the political environment	**.636**	.589
**Factor II: Career support**		
Helped you finish assignments/tasks or meet deadlines that otherwise would have been difficult to complete		**.875**
Given or recommended you for assignments that increased your contact with higher level managers		**.810**
Gone out of his/her way to promote your career interests		**.769**
Given or recommended you for assignments that required personal contact with managers in different parts of the company		**.765**
Given or recommended you for challenging assignments that present opportunities to learn new skills		**.720**
Served as a role model		**.683**
Eigenvalue	12.291	1.496
Variance (%) (Total: 76.593%)	68.285	8.308
Kaiser-Meyer-Olkin MSA	.939	
Bartlett’s test of sphericity	4283.534**	
Reliability (Cronbach’s alpha)	.970	.924

*Note: N* = 195; **p < .01; Items are grouped for presentation purpose. Bolded loadings indicate the inclusion of those items in the factor.

**Table 3:** **Bases of power: rotated factors, item loadings, and reliabilities**

	Factors				
Items	I	II	III	IV	V
**Factor I: Personal power**					
He/she can make me feel personally accepted	**.822**				
He/she is a likeable person	**.799**				
He/she can make me feel important	**.794**				
He/she can make me feel like he/she is approved of me	**.786**				
He/she can make me feel valued	**.779**				
He/she can share his/her considerable experience and/or training with me	**.772**				
He/she possesses or has access to information that is valuable to others	**.772**				
He/she can use logic to convince his/her co-workers	**.760**				
He/she can provide me with sound job-related advice	**.759**				
He/she can convince workers by explaining the importance of the issue	**.758**				
He/she can provide me with good technical suggestions	**.744**				
He/she has the knowledge required for the job	**.737**				
He/she can provide me with needed technical knowledge	**.712**				
He/she can provide sufficient information to support my view	**.675**				
He/she can explain the reasons for his/her request	**.624**				
**Factor II: Legitimate power**					
He/she can make him/she recognizes that he/she has tasks to accomplish		**.839**			
He/she can make me feel like I should satisfy his/her job requirements		**.820**			
His/her position in the organization provides him/her with the authority to direct their work activities		**.808**			
He/she can make me feel that I have commitments to meet		**.758**			
He/she can give me the feeling that I have responsibilities to fulfill		**.756**			
**Factor III: Reward power**					
He/she can provide me with special benefits			**.841**		
He/she can increase my pay levels			**.803**		
He/she can give special help and benefits to those who cooperate with him/her			**.795**		
He/she can influence I get a promotion			**.746**		
He/she can influence whether I get a pay raise			**.673**		
**Factor IV: Coercive Power**					
He/she can give me undesirable job assignment					
He/she can make things unpleasant for him/her in his/her workplace				**.953**	
He/she can make work difficult for me				**.911**	
He/she can make being at work distasteful for him/her				**.909**	
He/she can administer sanctions and punishment to those who do not cooperate with him/her				**.903**	
**Factor V: Connection Power**					
He/she has connections with influential and important persons					**.786**
He/she has a lot of connection with others outside the organization					**.739**
He/she knows a number of influential people	.514				**.700**
He/she maintains close ties with powerful others within the organization	.513				**.686**
He/she is in good terms with top people within the organization	.519				**.616**
Eigenvalue	18.887	3.786	2.576	1.748	1.143
Variance (%) (Total: 80.40%)	53.964	10.817	7.360	4.993	3.266
Kaiser-Meyer-Olkin MSA	.927				
Bartlett’s test of sphericity	9189.580**				
Reliability (Cronbach’s alpha)	.973	.956	.925	.955	.954

*Note: N* = 195; **p < .01; Items are grouped for presentation purpose. Bolded loadings indicate the inclusion of those items in the factor.

On the other hand, the 5 extracted factors of power. Factor I which included 5 referent power items, 5 expert power items, and 5 information power items was named as “referexpertinfo” which was actually personal power. Factor II which included 5 legitimate power items was named as “legitimate”. Factor III which included 5 reward power items was named as “reward”. Factor IV which included 4 coercive power items was named as “coercive”. Factor V which included 5 connection power items was named as “connection”.

Tables 4 and 5 illustrated the 10 items of job stressors and 17 items of stress arousal which were exposed to a varimax rotated principal components factor analysis to assess the dimension of this dependent variable. The 2 extracted factors of job stressors were renamed as “role ambiguity” and “overload” respectively. Whereas, for stress arousal, Factor I was named as “discord” and Factor II was named as “relax”.

**Table 4:** **Job stressors: rotated factors, item loadings, and reliabilities**

Items	Factors	
	I	II
**Factor I: Role ambiguity/lack of control**		
Not knowing what your supervisor thinks of you, how he/she evaluates your performance	**.888**	
Not knowing just what the people around you expect of you	**.843**	
Being unclear on just what the scope and responsibilities of your job are	**.806**	
Feeling unable to influence your immediate supervisor’s decisions and actions that affect you	**.786**	
Not knowing what opportunities for advancement or promotion exist to you	**.661**	.501
Feeling that you have too little authority to carry out the responsibilities assigned to you	**.645**	.558
**Factor II: Overload**		
Feeling that your job tends to interfere with your family life		**.886**
Thinking that you’ll not be able to satisfy the conflicting demands of various people over you		**.868**
Thinking that the amount of work you have to do may interfere with how well it gets done		**.823**
Feeling that you have too heavy work load, one that you can’t possibly finish during an ordinary day		**.816**
Eigenvalue	6.893	1.081
Variance (%) (Total: 79.737%)	68.926	10.811
Kaiser-Meyer-Olkin MSA	.902	
Bartlett’s test of sphericity	1963.157**	
Reliability (Cronbach’s alpha)	.931	.940

*Note: N* = 195; **p < .01; Items are grouped for presentation purpose. Bolded loadings indicate the inclusion of those items in the factor.

**Table 5:** **Stress arousal: rotated factors, item loadings, and reliabilities**

Items	Factors	
	I	II
**Factor I: Psychological discord**		
Feeling sad or depressed	**.947**	
Preoccupied with recurrent thoughts	**.942**	
Upset	**.941**	
Feeling tense	**.940**	
Thinking about things that upset you	**.930**	
Annoyed	**.929**	
Concerned or worried	**.927**	
Having difficulty relaxing	**.923**	
Irritable	**.919**	
Having difficulty adjusting or just coping	**.909**	
Anticipating or remembering unpleasant things	**.902**	
Feeling frustrated	**.890**	
Repeating unpleasant thoughts	**.888**	
**Factor II: Relaxation**		
Feeling calm		**.979**
Feeling satisfied		**.974**
Feeling peaceful		**.961**
Feeling relaxed		**.953**
Eigenvalue	11.296	3.631
Variance (%) (Total: 87.805%)	66.445	21.360
Kaiser-Meyer-Olkin MSA	.944	
Bartlett’s test of sphericity	5562.798**	
Reliability (Cronbach’s alpha)	.986	.979

*Note: N* = 195; **p < .01; Items are grouped for presentation purpose. Bolded loadings indicate the inclusion of those items in the factor.

Table 6 illustrates the intercorrelations among the subscales obtained using Pearson correlation to determine whether the subscales were independent measures of the same concept. Generally, intercorrelations among the power dimensions registered value of between .20 to .77 (*p < .01*), whereas, the intercorrelations for the subscales of dependent variables, namely role ambiguity/lack of control, overload, psychological discord, and relaxation ranged from -.213 to .740 at the level of *p < .01.* On the whole, the results have demonstrated acceptable levels of correlation. As shown in Table 6, the standard deviations of the variables were either close to or exceeded 1.0, indicating that the study variables were discriminatory.

**Table 6:** **Correlation analysis: pearson correlation matrix**

	1	2	3	4	5	6	7	8	9	10	11
1. Psychosocial support											
2. Career support	.768**										
3. Personal power	.448**	.340**									
4. Legitimate power	.254**	.192**	.668**								
5. Reward power	.165*	.195**	.617**	.596**							
6. Coercive power	.121	.172*	.199**	.296**	.253**						
7. Connection power	.499**	.408**	.801**	.589**	.546**	.326**					
8. Role ambiguity/lack of control	.178*	.277**	.189**	.153*	.223**	.538**	.267**				
9. Overload	.341**	.362**	.330**	.240**	.202**	.414**	.367**	.740**			
10. Psychological discord	.087	.057	-.130	-.190**	-.108	.224**	-.031	.286**	.327**		
11. Relaxation	-.044	-.003	.137	.253**	.176*	-.009	-.008	-.162*	-.213**	-.165*	
No. of item	11	6	15	5	5	4	5	6	4	13	4
Mean	3.968	3.806	4.964	5.235	4.585	3.903	4.958	3.825	3.921	1.964	2.830
Standard deviation	1.327	1.269	.970	1.091	1.337	1.534	1.191	1.224	1.404	.958	1.244

*Note:* **Correlation is significant at the 0.01 level (2-tailed); *Correlation is significant at the 0.05 level (2-tailed).

A 3-step hierarchical multiple regression analysis was carried out to test the hypotheses that comprised the direct and moderating effects of power bases, mentoring, and job stress. Tables 7 and 8 present the results of the analyses of the three constructs.

**Table 7:** **Regression analysis on power bases and job stressors with the interaction effect of mentoring**

	Job stressors					
Criterion variables	Role ambiguity/Lack of control			Overload		
	Std Beta (Model 1)	Std Beta (Model 2)	Std Beta (Model 3)	Std Beta (Model 1)	Std Beta (Model 2)	Std Beta (Model 3)
Predictor variables						
Personal power	.050	.043	.872*	.231	.189	.674
Legitimate power	-.145	-.125	-1.308***	-.059	-.035	-1.395***
Reward power	.098	.090	.509*	-.072	-.052	.532
Coercive power	.516***	.501***	.596**	.358***	.346***	.308
Connection power	.090	.041	.228	.139	.041	.613
Moderating Variables						
Psychosocial support		-.080	.899		.069	.158
Career support		.227*	-.435		.186	.106
Interaction Variables						
Personal power * career support			-2.929*			-2.420
Legitimate power * career support			.562			.977
Reward power * career support			2.792***			2.475***
Coercive power * career support			-.575			-.743
Connection power * career support			1.130			-.200
Personal power * psychosocial support			.980			1.243
Legitimate power * psychosocial support			1.606*			1.643
Reward power * psychosocial support			-3.397***			-3.397***
Coercive power * psychosocial support			.454			.837*
Connection power * psychosocial support			-1.214			-.655
*R* ^*2*^	.311	.338	.482	.247	.293	.429
Adjusted *R* ^*2*^	.293	.313	.433	.227	.266	.374
*R* ^*2*^ change	.311	.027	.144	.247	.046	.136
*F*	17.060***	13.633***	9.697***	12.383***	11.051***	7.823***

*Note: N* = 195; *p < .05, **p < .01, ***p < .001; *R* ^*2*^ = *R* ^*2*^ change for each step; Beta = Standardized beta coefficients.

**Table 8:** **Regression analysis on power bases and stress arousal with the interaction effect of mentoring**

	Stress arousal					
Criterion variables	Psychological discord			Relaxation		
	Std Beta (Model 1)	Std Beta (Model 2)	Std Beta (Model 3)	Std Beta (Model 1)	Std Beta (Model 2)	Std Beta (Model 3)
Predictor variables						
Personal power	-.077	-.122	.292	.177	.195	.024
Legitimate power	-.271**	-.269**	-.275	.309**	.313**	1.153**
Reward power	-.035	.004	-.139	.095	.076	-.187
Coercive power	.291***	.298***	-.228	-.038	-.045	-.562*
Connection power	.114	.055	-.397	-.371**	-.358**	-.246
Moderating Variables						
Psychosocial support		.214	1.736*		-.114	-.156
Career support		-.089	-2.365***		.097	.847
Interaction Variables						
Personal power * career support			-2.464			.315
Legitimate power * career support			.280			-.784
Reward power * career support			2.573***			-1.180
Coercive power * career support			.297			1.082*
Connection power * career support			2.632*			-.201
Personal power * psychosocial support			1.488			-.142
Legitimate power * psychosocial support			-.459			-.767
Reward power * psychosocial support			-2.428**			1.494
Coercive power * psychosocial support			.420			-.374
Connection power * psychosocial support			-1.143			-.097
*R* ^*2*^	.127	.145	.331	.122	.127	.204
Adjusted *R* ^*2*^	.104	.113	.266	.099	.094	.128
*R* ^*2*^ change	.127	.018	.185	.122	.005	.077
*F*	5.500***	4.537***	5.142***	5.270***	3.892**	2.668**

*Note: N* = 195; *p < .05, **p < .01, ***p < .001; *R* ^*2*^ = *R* ^*2*^ change for each step; Beta = Standardized beta coefficients.

### Regression analysis of power bases and job stressors with the interaction effect of mentoring

Table 7 illustrates the power bases with components of job stress namely, role ambiguity and lack of control. Table 3 illustrated power bases with components of job stress namely, role ambiguity and lack of control. Only one type of power namely coercive power was positively related with role ambiguity/lack of control (*p* < .001) and overload dimensions of job stressors. On the other hand, career support was found to have direct positively effect on role ambiguity/lack of control (*p* < .05). In step 3, mentoring was found to have moderated the relationship between reward power on both dimensions of job stressors (*p* < .001), personal and legitimate power on role ambiguity (*p* < .05), and coercive power on overload dimension of job stressors (*p* < .05).

### Regression analysis of power bases and stress arousal

Table 4 illustrates the relationship between power bases and stress arousal and mentoring. Legitimate power, coercive power were found to be positively related with psychological discord with *p* < .01 and *p* < .001, respectively. On the other hand, legitimate and connection power have positive significant effect on relaxation (*p* < .01). *R*^*2*^ value showed variance for direct effect explained 13 percent and 12 percent of the variability in psychological discord and relaxation dimensions of stress arousal. On the other hand, *R*^*2*^ value also indicated that the incremental variance explained 33 percent and 20 percent of the variability in psychological discord and relaxation dimensions of stress arousal. Mentoring was found to have moderated the relationship between power bases and dimensions of stress arousal. This indicated that the interaction effects of power bases and mentoring had significant contribution in explaining the variation in stress arousal.

In addition to that, we estimated the effect size using by following Cohen’s f^2^ procedure (Cohen 1988) and the formula given below:


*i* = interaction model

*m* = main effect model

The results suggest that for Psychological discord (*f*^2^ = 0.278), role ambiguity/lack of control (*f*^2^ = 0.278) and overload (*f*^2^ = 0.238) were of medium effect size while Relaxation (*f*^2^ = 0.097) was only a small effect size.

## Discussion

Overall, the stated research hypotheses received partial to moderate support from the data. As stated by Selvarajah and Denny (2008), managerial behavior is one of the important components associated with the excellent leadership in Malaysia. First, the statistical results have indicated a positive direct relationship between three dimensions of power, namely legitimate, coercive, and connection with job stress.

The analysis has indicated that position power such as legitimate power and coercive power were found to have direct effect on job stressors. This is because legitimate power is the target’s perception that the power holder has the authority or right to prescribe behaviour (French and Raven 1959). As evidenced in the past, position power can be used by managers to reward employees appropriately on their good performance and reduce their stress (Munir et al. 2012). Hence, leaders who possess legitimate power are able to give better instruction for the job of subordinates. Subordinates would tend to feel relax when carrying out the job since the instruction from leaders is not ambiguous. In addition, coercive power of the leaders was positively related to role ambiguity/lack of control, overload, and psychological discord. Coercive power which is commonly used among leaders (Sibley and Michie 1982; Stanworth 1984; Manaresi and Uncles 1995; Yavas 1998; Tourish et al. 2009) make subordinates unclear of their role as punishment without reasonable justification would lead to higher emotional distress of the subordinates (Lunenburg 2012). Besides that, the resulting analysis showed that connection power of the leaders was negatively related to relaxation dimension of stress arousal. Connection power means relationships, information networks, alliances, and communities of practice (Strang 2005). Leaders who have connection power would make their subordinates feel less tension. In addition to that, according to Turner (2005), people influence and control others through persuasion, authority, and coercion. As noted by past researchers, forcing tactics are even more effective when it is combined with non-forcing tactics. Mistakes in themselves can be costly but in addition there is the time taken to put things right. As stated by past researchers (e.g., Kamaruddin et al. 2012), certain level of stress, if it is managed properly, is constructive as it can help the employees to achieve better performance. Hence, the usage of a combination of various power are more effective than usage of a particular power on its own (Emans et al. 2003). Hence, hypothesis 1 is partially supported.

Interestingly, personal power such as referent power, expert power, and informational power were not found to have any effect on job stress. This could be due to the fact that in service industry, subordinates are well equipped with personal expertise, experience, training, and knowledge are likely to substitute leaders’ expertise, thus reducing their dependence on the leaders’ professional knowledge (Yagil 2002). As contended by Kipnis and Vanderveer (1971), personal power of supervisors would only have effect on low-power or incompetent subordinates and therefore, in this case, there is no need for supervisors to exert personal on subordinates. Hence, second hypothesis is rejected.

As hypothesized, mentoring was found to have moderated all types of power either one or two of dimensions of job stress, namely role ambiguity/lack of control, overload, relaxation, and psychological discord. This could be due to the fact that employers want to apply mentoring system to reinforce and enhance the reciprocal relationship with their subordinates. This is further supported by Eby et al. (2013) that mentors that are supportive to their subordinates, will build trust, intimacy, and interpersonal closeness would resulted in positive work and career attitudes, greater career success, and lower intentions to leave the organization among the employees. Hence, if there is apparent mentoring system, employees will be more committed. Mentoring does affect career success of the protégé in a positive manner and it was noted that individuals with mentors report more positive career outcomes than non-mentored individuals (Rajendran 2012).

As stated by Lee (2008), in an exchange characterized by trust and loyalty, leaders would delegate more challenging and relevant responsibilities that involve greater risk-taking to subordinates that they trust (Tierney and Farmers 2002). These findings can be explained by the theory of social exchange (Blau 1964) where employees would continue to commit themselves and stay with the organization if they are contented with the rewards based on their needs, expectations, desires or preferences (Chew and Chan 2008). As evidenced in the study by Salami (2010), mentoring plays a significant moderating role in the relationship between career plateauing and work attitudes. This is particularly true in high power distance country like Malaysia, as leading is a hierarchical relationship (Ansari et al. 2004) between subordinates who would tend to yield to superior authority and leaders who are expected to be paternalistic. Hence hypothesis 3 is partially supported.

## Implications

This research has a number of theoretical and practical implications both for scholars and practitioners, especially in the domain of Organizational Behavior. From a theoretical viewpoint, results of this study revealed the important link between power bases and the importance of having a good relationship between leaders and subordinates, and enhanced further the understanding of the employees’ job stress. Hence, this finding highlights the importance of power bases possessed by leaders as well as recognition of mentoring as a valuable approach for job stress. This study perhaps is the first that has systematically attempted to integrate power bases, mentoring and job stress in organizations.

Therefore, this study provides a conceptual foundation for the effective use of power bases. It has also enhanced understanding about the antecedent of power bases which subsequently resulted in a better knowledge of the employees’ job stress factors fundamental to employees’ work-related attitudes and behaviors. This study also extends extant research on the power bases, mentoring and employees’ job stress and hopefully stimulates the need for more research incorporating the perspectives of both parties.

## Limitations

In view of the fact that the supervisors and subordinates were mainly from service industry companies, hence different cultural and international contexts may limit the generalizability of results. Comparative studies across professions, cultures, and industries are needed in order to truly understand many of the constructs included in \this study. Clearly, this is an area that calls for further investigations. Next, this is not a longitudinal study; hence the direction of causality cannot be determined. Clearly, a longitudinal approach would have placed researcher in a better position to draw causal conclusions. Therefore, only conclusions or discussions of the general relationships between the variables of interest could be drawn. However, the current study makes an important contribution to the understanding of how power bases and mentoring could have significant effect on the use of employees’ job stress.

## Direction for future research

Though this study has contributed to the importance of power bases theory, yet future endeavors should be dedicated to comparing these findings with similar predictors and criterion in other sectors. All in all, this study suggests that managers in the service sector should seriously look into their power bases as it plays an important role in motivating and inspiring employees. In addition, this study did not examine the subordinates’ relationships with their supervisors as a potential influence on the supervisors’ usage of power bases. This is so because it is assumed that since supervisors are the key representatives of the organizations, their influence over their subordinates’ attitudes and behaviors would supercede that of their subordinates’ over them. Thus, another potential area for research is empirically testing the distinctions and the relationships between leader-member exchange and other constructs.

## Conclusion

This research is perhaps the first that contributes to management in general and how Malaysian leaders exert their power and its effect on employees’ job stress, with mentoring as an influence attempt. As stated by Lees (2014), managers must learn to diagnose how power could be obtained and wielded effectively in order to advance their goals. This study may be useful to those who are in positions of influence, to help the supervisors and subordinates understand more clearly the basis of their own actions, and the possible alternatives to their actions. Organizations that are serious about positive work outcomes should be more cognizant of the importance of applying effective power bases. This study has inevitably provided some empirical support to verify the notion that mentoring between supervisors and subordinates does play a role in moderating the effective use of power bases. It is believed that this study would have added value to the literatures on supervisors’ power bases, especially in the Malaysian settings since there is limited literature done in similar settings. Practically, this research points to the fact that Malaysian managers and executives need to be trained in the effective use of power bases.
